# Barriers and enablers to salt intake reduction in Australian adults with high blood pressure

**DOI:** 10.1017/S0007114524002174

**Published:** 2024-09-28

**Authors:** Saman Khalesi, Edwina Williams, David W. Johnson, Jacqui Webster, Abbie Fewings, Corneel Vandelanotte

**Affiliations:** 1 Appelton Institute & School of Health, Medical and Applied Sciences, Central Queensland University, Rockhampton & Brisbane, Australia; 2 Centre for Kidney Disease Research, University of Queensland, Brisbane, Australia; 3 Translational Research Institute, Brisbane, Australia; 4 Metro South Kidney and Transplant Services, Princess Alexandra Hospital, Brisbane, Australia; 5 The George Institute for Global Health, University of New South Wales, Sydney, Australia

**Keywords:** Salt, Sodium, Diet, Hypertension, Blood pressure

## Abstract

High dietary salt intake is a known risk factor for hypertension. However, Australians continue to consume excessive amounts of salt. The purpose of this study was to identify barriers, enablers and strategies to reduce salt in a sample of Australian adults with hypertension. This was a qualitative study. Participants were asked a set of open-ended questions during focus groups conducted between October 2020 and April 2021. Sessions were recorded and transcribed. Using an inductive approach, the transcript data from the focus groups were thematically analysed. This involved checking accuracy, becoming familiar with the data, coding responses based on questions, identifying themes through common patterns and validating themes by grouping similar questions that represented the data and study aim effectively. Thirty-one adults (55 % females) with high blood pressure participated in the focus group discussions. Participants demonstrated good knowledge of high blood pressure risk factors but lacked an understanding of recommended salt intake levels and sources of hidden salt. Challenges in reducing salt intake included the limited availability of low-salt commercial foods. Participants suggested improved food labelling and the use of technology-based interventions to promote healthier choices. Findings highlight the need for behavioural interventions, policy reforms and collaborations between the government, food industries and health organisations to address high salt intake in the population.

Hypertension or high blood pressure affects one-third (34 %) of Australian adults^(1)^. It adds to the burden of health care, including $4·52 billion for hospitalisations and $1·68 billion for prescriptions annually^(2)^. More than half of those with hypertension don’t have their blood pressure under control^(1)^. Uncontrolled blood pressure is a major risk factor for CVD, heart failure, chronic kidney disease, stroke and premature death^(2)^, further increasing the risk of morbidity and mortality and adding to the burden on the healthcare system.

Hypertension is a product of multiple factors, but diet plays a major role in the development and management of hypertension^(3)^. A diet that is consistently high in salt (sodium) can alter the fluid regulatory system and vascular resistance leading to increased blood pressure^(4)^. Therefore, the WHO along with all major health organisations globally recommends less than 5 g of salt intake each day to reduce the risk of hypertension and CVD^(5)^. There is a linear relationship between sodium intake and the risk of hypertension^(6)^. With every 2·5 g increase in salt (∼ 1 g of sodium), the risk of CVD increases by 6 %^(7)^. If a high salt intake is combined with high fat (especially saturated and trans-fat, which is predominant in processed and junk foods) and low fruit and vegetable intake, it can lead to a ∼2·5 times higher risk of high blood pressure compared with a healthy diet^(8)^.

Australians on average consume almost twice the recommended limit of salt^(8,9)^. Despite understanding the risk of high salt intake^(8,10)^, even Australians with known hypertension continue to consume a high amount of salt^(8)^. A number of barriers may impact high salt intake behaviour at the individual level (e.g. not knowing the high sources of salt/sodium in food, taste preference, lack of cooking skills, poor label reading skills, etc.) and environmental level (e.g. high sodium commercial foods, poor labelling, etc.). These barriers have been explored previously in Australia at the general population level^(11,12)^. However, given that salt intake reduction is crucial to prevent and manage hypertension, it is important to explore barriers and challenges of lowering salt intake and strategies to overcome these barriers in this population. Also, advances in technology have increased the numbers and accessibility of applications and websites with a focus on dietary behaviours, cooking skills and food choices. In a recent cross-sectional study in Australia, more than half of the participants reported using diet-related apps and websites^(13)^. If acceptable, strategies using technology have the potential to effectively reach a wider population and engage appropriate target groups at minimal cost^(14)^, helping reduce salt intake at the population level.

To date, only a few countries have developed comprehensive national policies to reduce salt intake^(15)^ and address WHO’s global target of 30 % salt intake reduction at the population level by 2025^(16)^. Australia has not developed a national policy to reduce salt intake. Some interventions have aimed to reduce salt intake at the Australian general population level^(17–19)^. However, the success of these initiatives has been modest to date. Therefore, this study aims to further add to the literature by identifying barriers and enablers to reducing salt intake in a sample of Australian adults with hypertension and exploring strategies and attitudes towards the use of technology to lower salt intake. The findings of this study will guide the development of public policies and behaviour change interventions to reduce salt intake and prevent and manage hypertension.

## Methods

### Design

Australian adults with hypertension (or high blood pressure) were invited via Facebook advertising, email to CQUniversity staff and students or by word of mouth to take part in online (using Zoom video conferencing software) focus groups. The invitation directed potential participants to the Salt Education (SaltED) webpage (www.salted.org.au) where further information relating to the study was located. Eligibility was confirmed using four questions based on age (18 years and older), self-reported blood pressure measurements (based on the latest reading at home or by a health practitioner), hypertension status (hypertension medically diagnosed by a health practitioner) and current self-reported medication to lower blood pressure. Participants who met the eligibility criteria and had internet access and online video conferencing ability were invited to complete the consent form. Participants were then directed to complete a short anonymous pre-focus group survey developed using the Qualtrics platform and designed to collect socio-demographic information, age, height, weight, hypertension diagnosis and medication and knowledge of salt. Participants were asked to identify the health behaviours that lead to high blood pressure. To assess the basic level of salt and blood pressure knowledge, participants were asked about the risk factors of hypertension, the daily recommended maximum intake of salt, how much salt they think they consume daily and to identify foods high in salt from a list of options provided.

Focus groups were conducted in groups of between six and twelve participants. This group size was chosen to encourage discussion but not be difficult to control^(20,21)^. Focus group sessions were conducted between 27 October 2020 and 8 April 2021. They lasted for approximately 60 min each. Two facilitators (SK and EW) attended all sessions. At the beginning of each session, participants were asked to introduce themselves using only first names. Following introductions, a facilitator outlined the structure of the session. Participants were then asked a set of open-ended questions developed based on the aim of the study (Table 1). Prompts were made by facilitators if needed. Focus group sessions were continued until no new concepts about the topic were emerging and further discussion added no benefit to the research^(21)^. Sessions were recorded and transcribed by an independent transcription agency (Rev.com; San Francisco). Two researchers reviewed and validated the transcriptions. At the end of each session, participants were provided with a supermarket gift card to the value of $20 for their participation. Ethics approval was obtained from the CQUniversity Human Research Ethics Committee (approval no. 0000022350) prior to the study.

**Table 1.** Main focus group questions

1	What do you think are the major sources of salt in the diet?
2	What actions (if any) are you taking to lower your salt intake?
3	What do you think are the barriers or challenges to lower salt intake?
4	Do you have/can you suggest strategies or actions to overcome these challenges and reduce your salt intake?
5	Do you think technology like smartphone apps or websites that provide tips on choosing foods and low-salt recipes can help you lower your salt intake?
6	What do you like and dislike about these technologies?
7	What should a website/app include to help you manage your salt intake?
8	An online/virtual salt coach intervention can provide personalised advice. In order to give this feedback, would you be happy to provide information such as age, sex, health status, current salt intake, interests, barriers and challenges to reduce salt?

### Data analysis

An inductive approach^(22)^ was used to thematically analyse transcription data. First, two researchers (SK and EW) checked the transcripts for accuracy against original recordings and became familiar with the data. Second, the transcript data from each focus group were exported to Microsoft Excel and sorted by question. Grouping all answers to each question provided the initial codes based on the questions. Third, themes were identified by searching for common patterns that emerged in each initial code. Fourth, the identified themes were reviewed and validated by further grouping similar questions to themes that represented the data and the aim of the study coherently. Throughout the focus group data interview, data collection and analysis and reporting processes of the study, the researchers adopted reflexive practices^(23)^. The researchers involved in this study are experienced in nutrition and behaviour change therapies and chronic disease management and contributed to the design of the study and interview questions. However, researchers continuously explored their positioning by reflecting on participants’ comments and preferences and discussed newly emerged ideas within focus groups to develop deeper understanding and knowledge.

## Results

Thirty-one adults (55 % females) with high blood pressure participated in the focus group discussions. Characteristics of the participants are outlined in Table 2. The average age of participants was 51 years (ranging from 24 to 77 years of age). Most participants in this study (71 %) were either in full-time, part-time or casual employment. A high number of participants had either completed technical studies (TAFE) (26 %) or university studies (68 %). Nearly all participants (77 %) were diagnosed with hypertension over a year prior to attending the focus group sessions. The average systolic blood pressure was 135 mmHg (lowest 105 mmHg and highest 180 mmHg) with the average diastolic blood pressure being 88 mmHg (lowest 70 mmHg and highest 110 mmHg). Many participants reported taking medication (65 %) to control their blood pressure. Participants reported an overall average BMI of 30 kg/m^2^ (range 22 kg/m^2^ to 42 kg/m^2^).

**Table 2.** Participant characteristics (Numbers and percentages; mean values and standard deviations)

Participant characteristics	*n*	%
Total participants *n*	31	
Males	14	
Females	17	
Education *n* (%)		
Secondary school	2	6
Technical studies (TAFE)	8	26
University studies	21	68
Employment *n* (%)		
Employed full-time	19	61
Employed part-time/casual	3	10
Pensioner	1	3
Retired	4	13
Student	1	3
Unemployed	3	10
Shift work *n* (%)		
Work shifts	2	6
Do not work shifts	29	94
Income *n* (%)		
Less than $30 000	7	23
$50 001–$70 000	2	6
$70 001–$100 000	9	29
$100 001–$150 000	6	19
> $150 000	7	23
Hypertension diagnosis length *n* (%)		
< 3 months	2	6
3–6 months	1	3
6 months to a year	4	13
> a year	24	77
Medication *n* (%)	20	65
	Mean	s d
Self-reported blood pressure		
Systolic blood pressure (mmHg)	135	15
Diastolic blood pressure (mmHg)	88	11
BMI (kg/m^2^)	30	6

### Knowledge of hypertension risk factors and salt intake

Participants correctly identified the major risk factors of high blood pressure as obesity (97 %), a diet high in salt (94 %), physical inactivity (90 %), high alcohol consumption (77 %) and tobacco smoking (74 %). However, just under half (48 %) of the participants were able to correctly identify the recommended maximum total daily intake of salt (5 g) for adults to reduce blood pressure and the risk of heart disease.

From the list of food options provided, bread, cheese, crackers, processed meats, canned soup, chips and gravy/sauces were food sources high in sodium^(24)^. However, only 23 % of participants correctly identified ‘breads’ as a major source of sodium, and less than one-third identified ‘cheese’ (13 %) and ‘crackers’ (29 %) as major sources of sodium. ‘Potato chips’ were correctly identified as a high salt source by almost all (97 %) participants. All 31 (100 %) participants identified fresh fruits such as ‘apples’ as low-salt food items.

### Attitudes, barriers and enablers of salt intake reduction

Focus group discussions identified four main themes around salt intake reduction: (i) major dietary sources; (ii) barriers and challenges; (iii) suggested strategies and actions; and (iv) attitude about using technology to help. A summary of the qualitative themes is outlined in Table 3.

**Table 3.** Thematic analysis of participant’s responses

Main codes	Themes
Major dietary source of salt	Added salt (cooking/table)
	Fast food and restaurant food
	Processed and packaged foods
Barriers and challenges	Availability of healthier commercial food choices and hidden salt
	Habit, taste and culture
	Lack of time
	Complex food labels
Suggested strategies and actions to lower salt	Self-help resources
	Salt alternatives
	Mindful eating
	Peer support
	Better nutritional information on commercial restaurant foods
	Advice and guidelines from reputable health organisations
Attitude about using technology to help reduce salt intake	Trust (information sharing, information accuracy)
	Motivation to use apps and websites (user-friendly, applicable information, peer/expert support and goal-tracking feedback)
	Desired functionality (applicable recipes, personalised information, help with food choices) Undesired functionality (too many notifications, time-consuming)

### Major dietary source of salt

Participants identified added salt (cooking/table), fast food and restaurant food along with processed and packaged foods as the major dietary sources of salt in their diet. Participants were generally aware of the consumption of salt through these sources. Many participants reported that they add salt to cooking or at the table, suggesting that ‘I put salt on everything…’, ‘for cooking some foods like steak, fries or tomato it is necessary…’, ‘I add salt to fresh fruits like oranges and apples that have tangy taste…’.

Participants also acknowledged that ‘restaurant foods, takeaway or dine-in foods generally tend to have a high salt content’ but also noted that the level of salt in these foods is usually unknown but high. Processed and packaged foods were also identified as major sources of dietary salt. Snacks, deli foods, frozen packaged foods, vegemite (savoury spread of concentrated yeast extract high in salt) and chips were suggested as major sources of dietary salt. Participants reported that ‘processed foods, especially from the deli’ such as ‘meats are usually processed in salt’ and that ‘they [food industries] try to bring out the flavours to attract people to buy their products’. Therefore, ‘packaged and frozen [foods]’, ‘sausages’ and ‘even some breakfast cereals’ are high in salt.

### Barriers and challenges

The limited availability of low-salt commercial food choices was suggested as a barrier to reducing salt intake. ‘Every packaged food has a lot of salt in it’, and many foods purchased commercially have salt that is ‘not visible’. ‘Restaurants chuck a lot of salt on steak and anything they cook’. ‘Every food item, apart from fruit and vegetables, has salt’.

Habits, tastes and cultural norms were also suggested as barriers to reducing salt intake. Participants reported that they ‘like the taste of salt’ and ‘vegetables are very boring without salt’. They also acknowledged that the environment plays an important role. ‘My grandmother added salt to literally everything [when cooking] and then added salt on top of that [when eating]’; ‘our culture is very salt-based, especially during festive season, it is very difficult to control my salt’; ‘I sometimes don’t add salt during cooking but getting those meals over the line with my teenage boys is a struggle’; and ‘socially eating out with friends is common in my life and it is difficult to avoid salt, sugar and butter [when doing so]’.

A lack of time for preparing healthy meals or choosing healthier food options is another barrier identified by participants. They suggested that having kids ‘keeps them very busy’ and ‘getting home late and have time to plan and cook [a healthy] dinner is a challenge’, and ‘sometimes it takes longer to prepare good healthy foods than reaching for a jar of something [ready-made, commercial]’. They also suggested that complex food labels add to the lack of time challenge, explaining that ‘labels are complicated… with so many different labelling systems’ and are ‘difficult to understand’. They also suggested that they are sometimes difficult to read ‘if you don’t have your glasses’ and that it takes a lot of time to ‘read through the label then convert that back [to serves]’.

### Suggested strategies and actions to lower salt

Some individual and environmental strategies were suggested by participants to help lower salt intake. At the individual level, self-help resources, salt alternatives and mindful eating and food choices were suggested. Self-help resources, including ‘recipes from health organisations’ or ‘self-research’ to help cook healthier and ‘using apps like MyFitnessPal’, ‘something that is easy to use’ or ‘barcode scanning’ to help ‘rank everything’ based on salt/sodium content, were suggested. ‘Planning meals’, ‘making food from scratch’, ‘studying food labels’ and using ‘herbs and spices’ as alternatives to salt were also suggested to help with mindful eating.

At the environmental level, peer support, better nutritional information on commercial and restaurant foods and medical advice and guidelines from reputable health organisations were suggested to help with lowering salt intake. ‘Having a partner in crime that is on board with you really helps’. This was especially emphasised by participants who needed to prepare food for the rest of the family and suggested that ‘having to prepare something different for the kids and hubby and myself at each meal’ would make it very difficult to stick with healthier food choices. Also, they emphasised the role of a peer-to-peer or coach-to-peer support system that provides ‘a one-on-one coaching, to show how (we are) tracking… or a badge system suggesting you achieved [your goal] could help’. Another strategy suggested to help make better food choices and reduce salt intake was for the commercial food providers (food industries, restaurants, takeaways, etc.) to make the salt and sugar content of their foods or meals accessible. ‘If you had an app so that you could scan barcodes in supermarkets or food codes [in restaurants] and [reported back] like a traffic light system (red, amber, green) because it is high in sodium’ or ‘similar to the star rating system that is rated based on various levels [ingredients] like sugar and salt’ could help. Participants suggested that this could be a helpful strategy for fast food and restaurants to help consumers choose based on their dietary needs. They also suggested that governments could play a more direct role here to be more ‘legislative’ and mandate food providers to comply with recommended levels of salt (and other ingredients). Participants also emphasised the importance of strategies promoted by health organisations to educate the public and provide advice, tips and easy recipes for choosing healthier food options. ‘I did a program this year called… that is only in Queensland… it was excellent and there was no cost, so maybe getting the word out that these programs are available’. ‘I use the recipes of Diabetes Australia’, similar recipes provided by other health organisations could help with preparing foods that are healthy but also have flavour.

### Attitude about using technology to help reduce salt intake

Participants reported technologies such as apps and websites may be helpful in reducing salt intake depending on two main factors: trust and motivation.

Participants’ concern about the use of technology was directed towards the availability of their personal information online and the accuracy of the information they receive from these technologies. ‘I am concerned about my information being available online’, ‘the issue is what and with whom you are sharing your information’. But they also suggested that as long as they could share their information ‘anonymously and not their real name’ they have no issues sharing. Participants also suggested that they rather ‘take advice from a health professional than an app or website’. But if the technology is ‘endorsed by their doctor, dietitian or other health professionals or by reputable health organisations’, then they are more comfortable trusting them.

They also reported that motivation to use these technologies depends on several factors, including what they offer, how easy they are to use and the support and feedback they offer. They mentioned that most apps and websites ‘are very time-consuming’ and ‘add extra steps’, which can be barriers to using these apps. ‘I think they [apps and websites] rely on you being dedicated to like weighing all your food to track food and not for everyone’. They also suggested that technologies that can ‘convey health information in a fun light-hearted way’ that can offer enjoyable recipes tailored to different needs can be more motivating and engaging. ‘I want to see recipes that are for everyday normal people’ on ‘what [food] I’ve in my pantry’ and food that is ‘relevant and [those that I am] able to pick up on the Australian market’. Participants also suggested that they ‘would feel more accountable if someone checked in with them’, as a peer providing support or an expert who would follow up with their progress. Also, receiving feedback on their progress and goals was suggested as a feature that helps them engage with the programme and continue using the technology but also suggested that the feedback ‘needs to be comprehensive enough and personalised otherwise it could be referring to anybody and a waste of time’.

When probed into the desired functionality and features of such technologies, a number of preferred features were suggested. Applicable recipes for everyday meals that are healthy but also taste good, personalised nutrition education and advice tailored to their needs and the ability to help choose from commercial foods were among commonly desired features. ‘If [an app/website] gives alternatives for certain [food] choices, something that compares similar kinds of food with lower salt… I would definitely try that’. On the other hand, apps or websites that send ‘too many notifications’ or are ‘time-consuming’ were not preferred by most participants. ‘I’m already bombarded with notifications from so many different sources, having just another one would just be noise for me’.

## Discussion

This study has identified barriers, enablers and strategies to reduce salt in a sample of Australian adults with hypertension. Overall, participants had a good understanding of the risk factors of high blood pressure and correctly identified high salt intake, physical inactivity, high alcohol consumption and smoking as major risk factors. Knowledge of risk factors of chronic disease is generally high in Australia^(10)^. However, participants did not show a good understanding of the recommended level of salt intake to reduce the risk of heart disease. Similar findings were reported previously in a sample of Australian adults where just over a quarter of the participants correctly identified the maximum recommended daily salt intake^(10)^. Further, despite correctly identifying some major sources of dietary salt (i.e. processed and packaged foods), they were not able to identify sources, such as cheese and bread, as high sources of dietary salt. This may suggest the need for educational interventions and policies with a focus on the major sources of salt intake. Future interventions may need to identify local and commercial foods high in salt and provide examples of alternative lower salt options. Australia’s Healthy Food Partnership has achieved modest success in meeting reformulation targets for some commercial foods^(25)^. To be more effective, reformulation policies may need to set more stringent targets, closely monitor the implementation in high-salt food industries and offer strategies to help these industries reduce salt content^(25)^.

The availability of low-salt commercial and packaged foods was reported as a major challenge in reducing salt intake. A similar barrier has also been reported in the general population in Australia^(10)^ and the USA^(26)^ and food service providers in the USA^(27)^ and Korea^(27)^. Commercial sources of salt intake contribute to more than 75 % of total salt intake daily^(28,29)^. Governmental policies and strategies to reformulate foods have resulted in an overall 23 % reduction in the sodium content of commercial foods available in Australia between the 1980s to 2013^(30)^. However, this reduction has not been sustained as a more recent study suggested an increase in salt content of some packaged foods (flat bread, biscuits and bacon) between 2014 and 2019 in Australia^(31)^. Also, the salt intake of Australians is still around 9 g per d^(8,9)^, almost twice the recommended target of 5 g per d to reduce heart disease set by major health organisations^(5)^. Given the voluntary nature of the majority of the strategies suggested by the government, the food industry’s adoption of these strategies to reduce the salt content of commercial foods is slow. A study on UK salt reduction has reported resistance from food industries to reduce the salt content of commercial foods due to potential impact on taste, consumer acceptance, cost and sales^(32)^. Therefore, governmental policies to gradually reduce the salt content of foods to allow adaptation to lower salt taste may reduce the impact on consumer acceptance and sales and the resistance from food industries^(32)^. More intense collaboration between governments, food industries and food catering services, including restaurants, fast foods and chefs for mandatory salt reduction targets are required to improve the environmental barriers of high salt intake^(33)^. There is evidence that government-led policies and interventions have been effective in improving health behaviour (e.g. tobacco control in Australia and Canada^(34)^). However, there is also evidence that large food industries have a significant influence on governmental policies both directly by involvement in the policy-making process and indirectly through political donations and relationships, funding and generating scientific evidence^(35)^. Whilst governmental policies to lower salt intake rely on collaboration with food industries, the risk of influence and conflict of interest should be mitigated by having clear and strict policies surrounding such collaboration^(35,36)^.

Participants also suggested better food labelling as a strategy to help reduce salt intake. Food labelling in general has a positive impact on healthy intake, especially if they are easily located and understood^(37,38)^. Australia’s Health Star Rating system uses front-of-package labelling with a simplified starting system for the healthiness of the food and is relatively well-performed^(39)^. However, food industries are implementing this rating system voluntarily^(40)^ and mostly on products that score higher (healthier options) in Health Star Rating than those that score lower (less healthy)^(36,40)^. Moreover, the rating algorithm is limited to some key nutrients, and the public lack of understanding of the rating interpretation may lead to the misperceived healthiness of some ultra-processed foods^(38)^. Governmental policies to improve the comparability of the rating system and mandating the use of the system for all packaged foods may help with healthier food choices and lower salt intake in Australia.

At the individual level, it is also crucial to continue raising awareness, educating consumers and equipping them with tools to make healthier food choices that impact the industry supply of such foods. Educational interventions to improve nutrition knowledge and skills to choose healthier foods have been effective^(41–43)^. Participants with high blood pressure in this study also suggested self-help resources and education to improve skills to choose lower salt foods as strategies to reduce salt intake. They also suggested the use of engaging and trustworthy technologies such as dietary apps and websites to help choose lower salt food options. Technologies such as dietary apps and websites have the potential to reach a wider population at a lower cost to help improving dietary intake^(44–46)^. However, trust in the quality of the information provided is an important challenge in using apps, websites and other technologies to improve health^(13,47)^. Technologies with endorsement from health organisations and professionals are generally deemed more credible and trustworthy^(44)^ and may help with the wider adaptation of these technologies. Features such as tailoring and personalisation, social support, goal setting and self-monitoring improve motivation to use the technologies^(13,44)^. Similar findings were reported by the participants in the current study. Therefore, dietary apps and websites developed by experts and supported by health organisations with the ability to personalise their content to individual needs and goals have the potential to influence the salt intake of individuals at the population level.

To the best of the researchers’ knowledge, this study was the first to explore the barriers and enablers of salt intake reduction in a sample of Australian adults with high blood pressure. The findings of this study provided essential information for public policies and interventions aiming to reduce salt intake. However, this study also had limitations. In focus groups, often dominant participants prevent reserved participants from contributing^(21,48)^. While the moderators made every effort to engage everyone in the conversation, not all participants participated comparably. Also, the majority of the participants in this study were educated and employed, limiting the generalisability of the findings. Focus group studies allow participants to be self-selected^(49)^. Therefore, participants who participated in this study were likely to be more health conscious and interested in improving their health and well-being, thereby skewing the study’s findings. However, the literature suggests more educated individuals with a strong interest in health are more likely to engage in activities and interventions to improve health behaviours^(13,50,51)^. Therefore, future research may need to target those most vulnerable and less likely to engage in health behaviour modification to address health and nutrition inequalities. Technology-based interventions can efficiently reach a broader population at a lower cost and help mitigate health inequality. Also, larger population-based studies are required to assess the acceptability of the strategies suggested and to ensure that they target those most at risk before implementing any policy changes or public interventions.

Overall, the adults with high blood pressure who participated in this study appeared to have a good understanding of the general lifestyle factors associated with high blood pressure. However, various individual psychological and environmental factors, including lack of motivation and time, habits and tastes, complex labelling, limited availability of healthier commercial foods and lack of practical knowledge, restricted their ability to modify their health behaviour and make healthier food choices. Strategies such as self-help resources from reputable sources of information, peer support and personalised feedback and advice were suggested by the participants as means to overcome these barriers at the individual level. Technologies including dietary apps and websites may also help if they are tailored to individual needs and are trusted and user-friendly. At the environmental level, stringent policy reforms and collaborations between the government, food industries and health organisations are important for a successful reduction of salt intake in the population. Strategies and policies, such as reformulating ultra-processed foods^(52)^ and implementing a tax on ultra-processed foods or subsidies for healthier, minimally processed options^(53,54)^, may need to be mandated or legislated to increase the availability and affordability of healthier food choices and improve population health.
